# Longitudinal Neuropsychological Assessment of Symptomatic Edema after Subthalamic Nucleus Deep Brain Stimulation Surgery: A Case Series Study

**DOI:** 10.3390/neurolint16010004

**Published:** 2023-12-28

**Authors:** Silvia De Ieso, Giulia Di Rauso, Francesco Cavallieri, Daniela Beltrami, Alessandro Marti, Manuela Napoli, Rosario Pascarella, Alberto Feletti, Valentina Fioravanti, Giulia Toschi, Vittorio Rispoli, Francesca Antonelli, Annette Puzzolante, Giacomo Pavesi, Federico Gasparini, Franco Valzania

**Affiliations:** 1 Neurology Unit, Neuromotor and Rehabilitation Department, Azienda USL-IRCCS di Reggio Emilia, 42123 Reggio Emilia, Italy; silvia.deieso93@gmail.com (S.D.I.); giuliadirauso3@gmail.com (G.D.R.); daniela.beltrami@ausl.re.it (D.B.); alessandro.marti@ausl.re.it (A.M.); valentina.fioravanti@ausl.re.it (V.F.); giulia.toschi@ausl.re.it (G.T.); federico.gasparini@ausl.re.it (F.G.); franco.valzania@ausl.re.it (F.V.); 2 Clinical Neuropsychology, Cognitive Disorders and Dyslexia Unit, Neuromotor and Rehabilitation Department, Azienda USL-IRCCS di Reggio Emilia, 42123 Reggio Emilia, Italy; 3 Clinical and Experimental Medicine PhD Program, University of Modena and Reggio Emilia, 41126 Modena, Italy; 4 Neuroradiology Unit, Radiology Department, Azienda USL-IRCCS di Reggio Emilia, 42123 Reggio Emilia, Italy; manuela.napoli@ausl.re.it (M.N.); rosario.pascarella@ausl.re.it (R.P.); 5 Neurosurgery Unit, Ospedale Civile Baggiovara (OCB) Hospital, Azienda Ospedaliero-Universitaria of Modena, 41126 Modena, Italy; alberto.feletti@gmail.com (A.F.); annette.puzzolante@gmail.com (A.P.); pavesi.giacomo@aou.mo.it (G.P.); 6 Neurosurgery Unit, Azienda Ospedaliera Universitaria Integrata Verona, 37126 Verona, Italy; 7 Neurology Unit, Neuroscience Head Neck Department, Ospedale Civile Baggiovara (OCB) Hospital, Azienda Ospedaliero-Universitaria di Modena, 41126 Modena, Italy; vit.rispoli@gmail.com (V.R.); antonelli.f@gmail.com (F.A.); 8 Neurosurgery Unit, Neuromotor and Rehabilitation Department, Azienda USL-IRCCS di Reggio Emilia, 42123 Reggio Emilia, Italy

**Keywords:** edema, deep brain stimulation DBS, neuropsychological, subthalamic nucleus, STN-DBS

## Abstract

Severe non-infectious or non-haemorrhagic brain edema surrounding the electrode represents a rare complication of subthalamic nucleus deep brain stimulation (STN-DBS) surgery. The aim of this study is to report three patients with advanced Parkinson’s Disease (PD) who developed symptomatic brain edema after STN-DBS surgery treated with intravenous steroids with a specific profile of reversible cognitive alterations. Patients were both assessed with a comprehensive neuropsychological battery including attention, memory, visuo-spatial and executive tasks. They were also briefly assessed for emotional and behavioural alterations, and for possible limitations in the activities of daily living. Normative data for an Italian population were available for all neuropsychological tests. The patients were firstly assessed before the surgery (baseline) as soon as they became symptomatic for the post-surgery edema and a few more times in follow-up up to ten months. In all patients we observed the resolution of cognitive deficits within six months after surgery with the corresponding reabsorption of edema at brain CT scans. The appearance of post-DBS edema is a fairly frequent and clinically benign event. However, in some rare cases it can be very marked and lead to important clinical—albeit transient—disturbances. These events can compromise, at least from a psychological point of view, the delicate path of patients who undergo DBS and can prolong the post-operative hospital stay. In this setting it could be helpful to perform a brain CT scan in 2–3 days with the aim of detecting the early appearance of edema and treating it before it can constitute a relevant clinical problem.

## 1. Introduction

Subthalamic Nucleus Deep Brain Stimulation (STN-DBS) represents an effective treatment in advanced Parkinson’s Disease (PD) patients [1,2]. It has been widely shown that, compared to pharmacological treatment, STN-DBS is more effective in improving motor functions and the quality of life of patients with advanced PD, in both short- and long-term follow-up [1,2,3]. Particularly, in the long-term, STN-DBS allows a stable improvement of motor complications, tremor and rigidity but with a less relevant effect on axial symptoms (e.g., gait and balance symptoms, speech and swallowing troubles) and cognitive decline [4,5]. The most common surgical complications of STN-DBS include intracranial haemorrhages, electrodes malposition, infection, seizures and ischemic strokes [6,7]. Severe non-infectious or non-haemorrhagic brain edema surrounding the DBS electrode represents another rare complication of STN-DBS surgery [8,9]. Nevertheless, idiopathic peri-lesion edema (IPLE) is increasingly reported [10]. Its incidence and association with post-operative symptoms, however, are still poorly established and its understanding and management are still limited [10]. IPLE occurs with delayed (≥72 h post-surgery) hypodense areas on computed tomography (CT) and T2/FLAIR hyperintensity on magnetic resonance imaging (MRI) around the DBS lead without signs of haemorrhage or infection [10]. In symptomatic forms, its most common symptoms are confusion, disorientation, behavioural alterations, headache, neurological focal signs, seizures, language deficits [8,11]. However, it was observed that an increasing number of asymptomatic patients present IPLE. It is very difficult to estimate the real prevalence of this complication, because in the days following implantation patients are not subjected to systematic CT or MRI scan, even in the presence of mild disturbances which are considered a normal consequence of surgery, rather than focal suffering.

It is well known that cognitive impairment in PD is common even in the initial stages with a prototypical pattern of dysexecutive syndrome [12,13,14,15]. Executive functions include the essential skills to maintain independent behaviour in daily life: they are planning, initiation, the ability to shift attention, the ability to carry out multiple actions simultaneously [12,13,14,15]. Executive functions worsen as the disease progresses and some are related to non-motor symptoms or behavioural disorders [12,13,14,15]. The impairment of executive functions is classically associated with the dysfunction of the fronto-striatal circuit which, starting from the dorsal striatum and putamen, affects various interconnected circuits [12,13,14,15]. Memory, language and visual-spatial impairments may be present in PD. Episodic memory deficit is common early in PD and may represent a risk factor for cognitive decline; episodic memory is impaired in recall more than in consolidation, suggesting frontal dysfunction. Similarly, fronto-striatal dysfunction can explain the greater impairment in naming actions or verbal fluency for actions, even in early stages of the disease. Cognitive assessment must include a comprehensive examination and specific tests for individual functions for many reasons, including the decision about whether the patient should be considered for DBS. One of the most frequently used tests is the Montreal Cognitive Assessment (MoCA) [16] which is more sensitive than the Mini-Mental state examination (MMSE) [17] in highlighting very mild symptoms, because it includes executive tests. The evaluation of individual and specific functions is more controversial. For example, for the evaluation of deficits in spatial cognition, the use of at least one visuoperceptive test is recommended, such as the Benton’s Judgment of Line Orientation Test or the figure copy tests. An internationally validated battery should be created; even behavioural disorders, being common, must be carefully evaluated and possibly with questionnaires validated for this disease. Data about the possible cognitive profile and outcomes in PD patients who develop symptomatic edema after surgery are still lacking in the literature. However, considering this aspect can allow for linking apparently general disorders to a focal problem and, in the weeks following implant, to have a clinical element of monitoring to target drug treatment. As a second objective, a cognitive evaluation can document the reversibility or otherwise of the edema-induced dysfunction.

Here, we report three patients with advanced PD who developed symptomatic brain edema after STN-DBS surgery with a specific profile of reversible cognitive alterations. The patients were both systematically assessed with neuropsychological complete evaluations, to define the pattern of cognitive impairment and to assess the evolution of symptoms during time.

## 2. Methods

Consecutive PD patients who developed non-haemorrhagic symptomatic edema after bilateral STN-DBS surgery in the last three years at our centre were included.

### 2.1. Clinical Assessment before Surgery

All patients were evaluated before surgery in accordance with the Core Assessment Program for Surgical Interventional Therapies in Parkinson’s Disease (CAPSIT-PD) [18]. All patients also underwent a 3 Tesla brain MRI in order to evaluate the presence of significant white matter hyperintensities of vascular origin [4]. Moreover, all patients underwent a detailed neuropsychological assessment and were also screened for the presence of pathogenic variants in PD-associated genes [4].

### 2.2. Genetic Assessment

All patients were included in the “Rostock International Parkinson’s Disease Study” (ROPAD) study which allows to test for the presence of mutations in genes involved in monogenic forms of PD [19]. In detail, the genetic profile was obtained by testing the patients for 11 pathogenic or likely pathogenic LRKK2 variants and GBA sequences. If negative, a next-generation sequencing panel targeting 68 genes involved in PD was performed. In particular, the following genes were tested: ADCY5; ANO3; APP; ATP13A2; ATP1A3; C19orf12; CHCHD2; COX20; DCTN1; DJ1; DNAJC13; DNAJC6; FBXO7; GBA; GCDH; GCH1; GNAL; GNE; HPCA; KCTD17; KMT2B; LRRK2; MAPT; PANK2; PARK2; PDE8B; PDGFB; PDGFRB; PINK1; PLA2G6; PRKRA; Rab39B; SGCE; SLC19A3; SLC20A2; SLC30A10; SLC39A14; SLC6A3; SNCA; SYNJ1; TAF1; THAP1; TOR1A; VAC14; VPS13C; VPS35; XPR1; APOE; ATP9A; CNEP1R1; CTDNEP1; ELOVL7; FBXO47; GAK; GRN; LPIN1; LPIN2; LPIN3; MCCC1; MCOLN1; NPC1; POLG; PSEN1; PSEN2; Rab12; SNCB; SYN1; TARTDP [19].

### 2.3. STN-DBS Surgery

All patients underwent bilateral STN-DBS surgery. Surgical trajectories were calculated using preoperative brain MRI and brain CT after placement of a stereotactic frame. The placement of intracranial electrodes was preceded by intraoperative micro-recording and micro-stimulation through recording microelectrodes (from three to five per side). The final quadripolar electrodes and the pulse generator were then placed (pectoral or abdominal region) under general anaesthesia. At the end of surgery, all patients underwent a brain CT scan to rule out the presence of complications related to the surgical procedure.

### 2.4. Neuropsychological Examination

Patients were both assessed with a comprehensive neuropsychological battery including attention, short-term memory, working memory, anterograde episodic memory, language, visuospatial, visuoconstructive and executive tasks. They were also briefly assessed for emotional and behavioural alterations, and for possible limitations in the activities of daily living. Normative data for an Italian population were available for all neuropsychological tests. The patients were firstly assessed before the surgery (baseline) as soon as they became symptomatic for the post-surgery edema and few more times in follow-up up to ten months. In particular, the following tests were included: Mini Mental State Examination [20]; CORSI block span forward/backward [21]; Phonemic and Semantic Fluency [22]; Raven’s Coloured Progressive Matrices; Frontal Assessment Battery [23]; Clock Drawing Test; Rey’s Figure Copy and Recall Test [24]; Apples Cancellation Test [25]; Visual Attention Test [26]; Trail Making Test [27]; Babcock Test [28]; Associate Learning [28]; Free And Cued Selective Reminding Test [29]; Visuo-Spatial Supraspan Test [30]; Benton’s Judgment of Line Orientation Test [31]; WAIS-IV Similarity Sub-Test [32]; Visual Naming Test; Time And Weight Estimation Task [33]; Modified Wisconsin Card Sorting Test [34]; Symbol Cancellation Test [35]; Letter Cancellation Test [35].

## 3. Cases Description

All patients following pre-operative screening were considered suitable for Deep Brain Stimulation surgery. None of them had significant cognitive impairment before surgery. Genetic analyses showed no pathogenic variants in PD-associated genes. Details of single clinical cases are presented as follows. The different results of the neuropsychological assessments are reported in the supplementary materials. The presence of an asterisk indicates a pathological value below the threshold of the specific test.

### 3.1. Patient 1

A 64-year-old female, right-handed, with 12 years of education was diagnosed with PD at the age of 54. PD started in the left side with hand rest tremor together with bradykinesia and rigidity.

**Figure 1:**
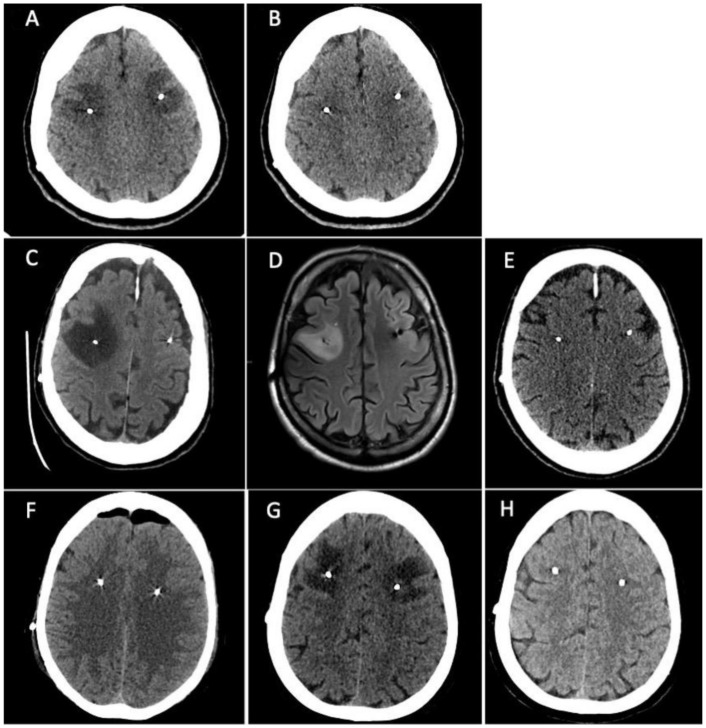
**Patient 1.** (**A**) Brain CT scan showed the presence of bilateral frontal subcortical edema surrounding the DBS electrodes. (**B**) a new brain CT scan showed the complete resolution of brain edema. **Patient 2**. (**C**) CT scan revealed the presence of a massive cerebral edema in right frontal lobe, surrounding DBS electrodes. (**D**) Brain MRI performed fourteen days later confirming the presence of a T2-weighted and FLAIR sequences hyperintense area in the right middle frontal subcortical lobe compatible with cerebral edema. (**E**) Brain CT scan repeated 30 days later showed the resolution of cerebral edema. **Patient 3.** (**F**) postoperative CT scan showed mild asymptomatic left frontal edema surrounding the homolateral DBS electrode. (**G**) Brain CT scan was repeated showing a great worsening of left middle-upper frontal edema that surrounded the DBS electrodes bilaterally, extending also to the left parietal lobe and near the right coronal suture. (**H**) Brain CT scan showed resorption of brain edema.

Treatment with pramipexole subsequently followed by levodopa was started with a good clinical response. Three years later the patient developed motor and non-motor fluctuations which led to the introduction of levodopa/carbidopa/entacapone followed by the appearance of peak-dose dyskinesia. Due to the worsening of motor and non-motor complications, the patient was admitted to our unit where she was preliminarily assessed for STN-DBS surgery. Baseline neuropsychological assessment did not reveal any cognitive deficits or emotional-behavioural alterations (Supplementary Table S1). Six months later she underwent bilateral STN-DBS surgery (Boston Scientific Gevia^TM^, directional leads) without any procedural complications seen at immediately postoperative CT scan. After five days from surgery the patient complained difficulties in language production (anomies, semantic paraphasia), spatial-temporal disorientation, and nocturnal wandering. Brain CT scan was repeated showing the presence of bilateral frontal subcortical edema surrounding the DBS electrodes (Figure 1A). The patient underwent a neuropsychological assessment which detected a cognitive worsening compared to pre-surgical cognitive baseline with the appearance of left unilateral spatial neglect related to the personal and peripersonal space (Apples Cancellation Task), executive deficits (Frontal Assessment Battery, Semantic Fluency, Raven’s Test, Corsi Blocks Span Backward) and a complete cognitive anosognosia (Supplementary Table S2). Treatment with intravenous dexamethasone (4 mg twice a day) was started and continued for one week with subsequent oral tapering in ten days. Three weeks after the surgery, a new brain CT scan showed the complete resolution of brain edema (Figure 1B) with an almost complete normalization of cognitive performances on neuropsychological assessment (Supplementary Tables S1 and S2). The left hemispatial neglect disappeared and the attentional-executive processes were more efficient. A middle and long-term neuropsychological assessment (two and ten months later, Supplementary Table S1) showed a cognitive profile which was almost equal to the pre-surgical one. All the scores were in the normal range, some of them below the mean. The emotional-behavioural alterations which appeared immediately post-surgery completely disappeared.

### 3.2. Patient 2

Patient 2 is a 55-year-old male, right-handed, with 17 years of education. He was diagnosed with PD at the age of 32 after the progressive onset of rigidity and bradykinesia in the right hand. Due to the presence of disabling motor complications with a severe worsening of dyskinesia and motor and non-motor fluctuations, the patient was admitted to our unit where he was preliminarily assessed for STN-DBS surgery. Baseline neuropsychological assessment showed the presence of slight executive and visuo-spatial deficits (Supplementary Table S3). Most of cognitive performances were normal. One month after cognitive baseline, he underwent the bilateral STN-DBS without complications (Boston Scientific Gevia^TM^, directional leads). Postoperative CT scan was negative. After five days the patient developed confusion with spatial-temporal disorientation, left inferior facial nerve palsy, dysphagia, and an unexplained loss of consciousness. A new CT scan was performed revealing the presence of massive cerebral edema in right frontal lobe, surrounding DBS electrodes (Figure 1C). Treatment with intravenous dexamethasone (4 mg twice a day) was started and continued for one week with subsequent oral tapering in ten days. Brain MRI was also performed fourteen days later confirming the presence of a T2-weighted and FLAIR sequences hyperintense area in the right middle frontal subcortical lobe compatible with cerebral edema (Figure 1D). Neuropsychological reassessment showed severe worsening of cognitive performances in the form of left unilateral spatial neglect (Apples Cancellation Task, Symbols Cancellation Test, Letters Cancellation Test) (Supplementary Table S4) and inhibitory control deficits (Frontal Assessment Battery, Visual Attention Test) (Supplementary Table S3). Patient underwent cognitive rehabilitation approximately for a month. After six weeks post-surgery, neuropsychological assessment was repeated revealing a significant cognitive and psychological improvement (Supplementary Table S3). However, the comparison with cognitive baseline assessment still showed a slight decline. Brain CT scan was also repeated 30 days later showing the resolution of cerebral edema (Figure 1E). Four months after surgery, neuropsychological assessment was repeated resulting completely negative. The patient underwent another neuropsychological assessment seven months after surgery which showed a slight worsening characterized by an isolated visuo-constructive deficit and under-mean performances in a few executive-attentive tasks.

### 3.3. Patient 3

A 58-year-old female, right-handed, with 12 years of education was diagnosed with PD at the age of 51. The clinical onset was at 46 years old in the left-body side with hand rest tremor, bradykinesia, and difficulties in fine hand movements. The family history was negative for movement disorders and genetic testing did not document any pathogenic variant in the main genes implicated in familial Parkinson’s disease. Treatments with pramipexole and safinamide were initiated, but subsequently discontinued due to being poorly tolerated by the patient. Therefore, levodopa was started with a good clinical response. Due to the worsening of motor and non-motor symptoms with motor fluctuation and peak-dose dyskinesia, the patient was preliminarily assessed for STN-DBS surgery. Baseline neuropsychological assessment did not reveal any significant cognitive deficits (Supplementary Table S5). Six months later she underwent bilateral STN-DBS surgery (Boston Scientific Gevia^TM^, directional leads) without any procedural complications seen at postoperative CT scan, except for mild asymptomatic left frontal edema surrounding the homolateral DBS electrode (Figure 1F). Six days after surgery, the patient complained of new onset procedural difficulties, particularly she reported the inability to line up actions to achieve a goal (e.g., sequences of actions for crocheting or for number dialling on the mobile phone). Brain CT scan was repeated showing a great worsening of left middle-upper frontal edema that surrounded the DBS electrodes bilaterally, extending also to the left parietal lobe and near the right coronal suture (Figure 1G). The patient underwent a second neuropsychological assessment which detected new executive deficits (Frontal Assessment Battery, Semantic Fluency, Corsi Block Test Backward, Raven’s Test), visuospatial deficits (Clock Drawing Test, Corsi Block Test Forward, Rey’s Figure Copy) and visuo-spatial neglect (Apples Cancellation Task) (Supplementary Tables S5 and S6). Treatment with intravenous dexamethasone (4 mg twice a day) was started and continued for one week with subsequent oral tapering in ten days. Seven days after the therapy onset, the patient reported a clinical improvement, and a new brain CT scan performed after three weeks showed the resorption of brain edema (Figure 1H). Seven months after surgery, neuropsychological assessment was repeated showing a cognitive profile almost superimposable to the pre-operative one (Supplementary Tables S5 and S6).

## 4. Discussion

In this article, we report a longitudinal extensive neuropsychological assessment in three patients who developed non-infectious, delayed-onset post-operative cerebral edema after bilateral STN-DBS surgery. Firstly, this case series study has important limitations which need to be underlined including the small sample size, the retrospective nature of the data and the difference in timing in postoperative neuropsychological assessments. In addition, we did not compare the cognition due to edema after STN-DBS to brain edema of other surgeries (i.e., meningioma surgery, or surgery for non-ruptured brain aneurysms).

In all patients the edema emerged after surgery and we observed a progressive cognitive improvement in the following weeks with the complete resolution of deficits within a range of 1–4 months (two months in patient 1, four months in patient 2, two months in patient 3, respectively), with a temporal correspondence with reabsorption of edema at brain CT scans. In the second case, due to the higher severity and diffusion of brain edema, the cognitive improvement was complete only four months after surgery. In all patients the cognitive normalization was confirmed in a long-term assessment at ten, seven and seven months, respectively. Several randomized studies (STN-DBS vs. best medical treatment) have not showed a worsening of the overall cognitive performance, assessed with the Mattis dementia scale, in the operated patients with a two-year follow-up [36,37,38]. However, STN-DBS has been found to negatively affect some specific cognitive domains. In particular, a postoperative decline in semantic and phonological verbal fluencies was found [39] which progressively worsens over time [40]. Furthermore, this finding was also found in patients operated on but not stimulated, leading to the hypothesis that the injury due to the passage of the electrodes represents the major cause of postoperative cognitive alterations [41]. However, it is believed that the pathogenesis of cognitive changes resulting from stimulation is more complex and is the result of the combination of several factors: stimulation of the cognitive-limbic area of the STN, post-operative pharmacological changes, passage of the electrodes and cognitive preoperative profile of the patient [42,43]. Advanced age, presence of attention deficit, high LEDD, high axial scores and low response to dopaminergic therapy represent pre-operative risk factors correlated with decline in cognitive performance in the post-operative phase [38,44]. A recent study has found that in patients with PD with longstanding STN-DBS, dementia prevalence and incidence are not higher than those reported in the general PD population. Except for few patients with perioperative cerebral haemorrhage, STN-DBS is cognitively safe, and does not provide dementia risk factors in addition to those reported for PD itself [45]. Recently, the role of genetic profile of PD patients treated with STN-DBS has gained attention particularly regarding the GBA gene. Indeed, two studies have confirmed the PD patients who carried heterozygous mutations in the GBA gene are at higher risk of developing cognitive alterations after surgery, even if the motor outcome of the surgery is good and maintained in the long-term [46,47]. The three patients that we have reported in this study were all negative for mutations in genes associated with PD including the GBA gene, excluding the possible presence of a genetic confounding factor.

Until now, only two studies in the literature have assessed cognitive function in PD patients who developed non-infectious, delayed-onset post-operative cerebral edema [48,49]. However, in both studies, cognitive function was assessed only through the Mini-Mental State Examination (MMSE), Montreal Cognitive Assessment (MOCA) and the Frontal Assessment Battery (FAB) [48,49]. However, since they did not assess the patients with an extensive neuropsychological assessment, it was not possible to detect subtle cognitive changes and to phenotype the neuropsychological pattern of STN-DBS delayed-onset post-operative cerebral edema. Concerning the mainly impaired functions, our cases all presented hemispatial neglect, a dysexecutive syndrome and visuo-constructional apraxia. This pattern of impairment is similar between the three patients, probably because the electrode’s entry areas are the same in all cases and consequently so is the perilesional edema. Furthermore, depending on the subsequent extension of this edema, the cognitive impairment has progressed to varying degrees of severity with possible involvement of further domains as well.

It is interesting to note that as reported by Nishiguchi et al., even in our three cases the severity and the diffusion of cerebral edema was associated with a greater cognitive involvement [49]. However, even if more severe, the cognitive impairment was transient with a complete return to preoperative condition.

Despite being a sample of only three patients, our report is in line with the findings of Sharma et al. [48], regarding the absence of cognitive alterations one year after cerebral edema confirming the safety of DBS procedure since the cognitive complications secondary to post-surgical edema appear transient.

The postulated pathophysiological mechanisms of non-infectious, delayed-onset post-operative cerebral edema include among others the possible use of irrigating solutions used during the surgical procedure; cerebral venous infarction; breakdown of the blood-brain barrier due to micro-haemorrhages or mechanical trauma; micro-lesions along the way for MER-guided implantation, and allergy [11]. However, the precise cause is yet to be elucidated [11,49].

A characteristic feature is the late onset of edema, usually 3–4 days after implantation. This suggests careful observation even when the patient presents an excellent recovery on the first day. A confounding factor could arise from the presence of a significant frontal pneumocephalus, which is often seen on CT performed at the end of surgery, to which the medical team could attribute the presence of mild cognitive impairment in the following days.

A recent study compared clinical and laboratory findings between patients (n = 61), who underwent DBS surgery, with IPLE (n = 23) and without IPLE (controls, n = 38) at delayed post-operative CT imaging [10]. Particularly, this study included patients with different underlying conditions, namely PD, dystonia, tremor and pain. Consequently, the targets included were not only limited to the STN, but also included the Globus Pallidus Internus (GPi), the Ventral Intermediate Nucleus (VIM), the Dorsal cingulate gyrus, the Ventralis Oralis Anterior and Posterior (Voa/Vop) nuclei of the thalamus and finally the caudal zona incerta. According to this study, the incidence of IPLE, based on routine delayed postoperative CT scan performed between 72 and 36 h after surgery, was 29.5% per electrode, and 37.7% per patient [10]. Moreover, the authors also reported that age, longer operative time and a greater number of microelectrode recordings (MERs) per electrode may represent risk factors for IPLE formation (although the last two conditions mentioned above did not reach statistical significance comparing patients with IPLE and controls) [10]. However, it is interesting to note that only two patients with IPLE (8.69% of the total IPLE cohort) presented with new onset neurological symptoms following surgery and received corticosteroid therapy (dexamethasone 4 mg two time daily slowly decreased within two weeks at discharge) [10]. In particular, none of symptomatic patients with IPLE reported new onset cognitive impairment and at the six-week follow-up neurological evaluation, neither of these two patients exhibited symptoms [10].

However, the incidence reported in the literature is uneven due to the absence of standardised routine post-operative imaging protocols [10]. Particularly, based on a literature review performed by Giordan et al. [10], IPLE incidence may be significantly underestimated if imaging is performed only on the basis of clinical and neurological changes, ranging from 3.1% to 6.1% [10].

A further recent study [50] analysed DBS complications and assessed potential predictive factors reviewing five hundred and seventeen consecutive cases of DBS for Parkinson’s disease performed between 2006 [50] and 2021 by the same neurosurgeon in a single centre. Four hundred and thirty-three of the surgeries had the subthalamic nucleus as target, 82 the postero- GPi, one the zona incerta (Zin), and one the ventralis intermedius medialis (VIM) nucleus [50]. Particularly, nine hundred and ninety-three leads were implanted in 517 procedures (832 of which were positioned in the STN, 158 in the GPi, two in the Zin, and one in the VIM) and in 126 cases (24.4%), directional leads were implanted [50]. According to this study, in 187 cases (36.2%), a postoperative 1.5 T MRI or a CT scan had revealed perielectrode brain edema [50]. However, only in 14 of these cases (2.7%) patients were symptomatic for edema, while in the remaining 173 cases (33.5%) post-operative brain edema was not symptomatic [50]. It is not specified, however, whether among the symptomatic patients anybody presented reversible cognitive impairment. Concerning potential predictive factors, in this study no association was found between the number of tracks used and the rate of brain edema [50]. Conversely, this research revealed different rates of edema between the three manufacturers of electrodes (Medtronic, Boston Scientific, and Abbott), with Medtronic in particular displaying the higher incidence (39.5%) compared to Boston Scientific (27.9%) [50]. Differently, the three patients in our case series had Boston Scientific directional electrodes. However, the increased incidence of perielectrode brain edema associated with certain lead models requires a future investigation. Finally, considering the two most utilized targets (STN and GPi), no significant differences were found in rates of brain edema in this study [50].

The treatment of non-infectious, delayed-onset post-operative cerebral edema is typically conservative, and it consists of intravenous or oral steroids. Edema usually tends to reabsorb itself in few weeks after the starting of steroid treatment, as in our cases [11]. Our message is that the appearance of post-DBS edema is a rather frequent and a clinically benign event.

However, in some rare cases it can be very marked and lead to important clinical—albeit transient—disturbances (seizures, dysphagia, etc.). These events can compromise, at least from a psychological point of view, the delicate path of patients who undergo DBS and can prolong the post-operative hospital stay.

## 5. Conclusions

In conclusion, based on our experience, in selected cases, a brain CT scan 2–3 days after surgery could be performed with the aim of detecting the early appearance of edema and treating it before it can constitute a relevant clinical problem. In addition, in more severe cases of edema, characterized by severe and longer-lasting cognitive deficits, it would be useful to carry out neuropsychological counselling to the patient and caregivers on how to manage cognitive disturbances in the ecological context, or in some cases, to carry out a brief accompanying cognitive rehabilitation.

## Supplementary Materials

The following supporting information can be downloaded at: https://www.mdpi.com/article/10.3390/neurolint16010004/s1, Table S1: Comparison between the different neuropsychological assessments (pre-surgical intervention, post-edema, follow-up) in patient 1. Table S2: Unilateral spatial hemineglect assessment in patient 1. Table S3: Comparison between the different neuropsychological assessments (pre-surgical intervention, post-edema, follow-up) in patient 2. Table S4: Unilateral spatial hemineglect assessment in patient 2. Table S5: Comparison between the different neuropsychological assessments (pre-surgical intervention, post-edema, follow-up) in patient 3. Table S6. Unilateral spatial hemineglect assessment in patient 3.
