# Supplementary material for: Longitudinal Neuropsychological Assessment of Symptomatic Edema after Subthalamic Nucleus Deep Brain Stimulation Surgery: A Case Series Study
Source: Neurol Int. 2023 Dec 28;16(1):62–73. doi: 10.3390/neurolint16010004 (PMC10801618; doi:10.3390/neurolint16010004)
Supplement: Supplementary file 1 [file neurolint-16-00004-s001.zip › neurolint-2610147-supplementary.pdf]

Supplementary Table S1. Comparison between the different neuropsychological assessments (pre-surgical intervention, post-edema, follow-up) in patient 1

	Pre-STN-DBS	Post-STN-DBS			
**Test**	**6 months**	**1 week**	**3 weeks**	**2 months**	**10 months**
**Screening**					
Mini-Mental State Examination (/30)	30	20*	27	/	29
**Attention and Working Memory**					
Visual Attention Test (/50)	48	26	42	/	39
Trial Making Test					
- Test A sec.	44	n.a.	/	/	34
- Test B sec.	95	n.a.	/	/	202
- B-A sec.	51	n.a.	/	/	168
**Short-term Memory**					
Digit Span Forward	5	5	5	5	7
Digit Span Backward	4	2*	3	3	4
Corsi Block Span Forward	5	3*	4	4	4
Corsi Block Span Backward	4	2*	4	4	4
**Verbal Long-term Memory**					
Babcock (/28)	14,5	10,5	/	15,5	21,5
Associate learning (/22,5)	9	/	/	/	15
FCSRT (/12)	12	9	/	/	/
**Visuo-Spatial Long-term Memory**					
Rey Figure – differed recall (/36)	15,5	11	10	/	13,5
Visuo-spatial Supraspan					
- Immediate recall (/29,16)	7,1	/	/	/	n.a.
- Differed recall (/1,62)	0,53	/	/	/	n.a.
**Visuo-spatial Functions**					
Rey Figure - copy (/36)	35	22	35	/	32
Judgment of Line Orientation Test (/30)	15	n.a.	/	16	19
Clock DrawingTest (/10)	/	0*	1*	10	
VOSP					
- Pre-test (/20)	19	/	/	/	19
- Incomplete Letters(20)	20	/	/	/	19
- Silhouettes (30)	24	/	/	/	23
- Object Decision (/20)	17	/	/	/	18
- Progressive Silhouette (/20)	8*	/	/	/	11*
- Dot counting (/10)	10	/	/	/	10
- Position Discrimination (/20)	20	/	/	/	19
- Number Location (/10)	6*	/	/	/	6*
- Cube Analysis (/10)	9	/	/	/	9
**Language**					
Phonemic Fluency	49	20	33	/	49
Semantic Fluency	48	26*	39	/	46
WAIS IV - Similarity (/36)	26	17	/	/	15
Visual Naming					

- Not Livings (/32)	32	30	/	/	32
- Livings (/32)	32	30	/	/	27
**Executive functions**					
Raven CPM 47 (/36)	33	15*	/	/	29
FAB (/18)	18	8*	16		18
Stroop					
- Time interference sec.	20,5	n.a.	/	28	19,5
- Error interference (/30)	0	n.a.	/	0	0
Time And Weight Estimation Task (STEP)					
- Time total (/30)	21	21	/	20	25
- Weight total (/30)	21	11*	/	13*	24
- Time + Weight total (/60)	42	32*	/	33*	49
- Time – Weight total (/60)	0	10*	/	7	1
Modified Wisconsin Card Srting Test - MCST					
- Categories (/6)	6	/	/	/	6
- Errors (/47)	7	/	/	/	2
- Perseverative Errors (/47)	4	/	/	/	1
**Mental health screening**					
Hospital Anxiety and Depression Scale - HADS					
- Anxiety (/21)	6	/	/	/	7
- Depression (/21)	6	/	/	/	8
Activities of Daily Living					
- ADL (/6)	6	/	6	/	6
- IADL (/8)	8	/	7	/	8

Abbreviations: Activities of daily living (ADL); Instrumental activities of daily living (IADL); frontal assessment battery (FAB).

Supplementary Table S2. Unilateral spatial hemineglect assessment in patient 1

Test	post edema STN-DBS	3 weeks later
**Apples Cancellation Task**		
Left Omissions (/20)	14	0
Right Omissions (/20)	0	0
Accuracy (total Omissions)	15*	0
Egocentric Asymmetry (/20)	14*	0
Total False positives	3	0
Allocentric Asymmetry (/20)	1	0
Total time (sec.)	250	100

Supplementary Table S3. Comparison between the different neuropsychological assessments (pre-surgical intervention, post-edema, follow-up) in patient 2

Pre-STN-DBS		Post-STN-DBS			
**Test**	**6 months**	**1 week**	**6 weeks**	**4 months**	**7 months**
**Screening**					
Mini-Mental State Examination (/30)	29	25	30	/	29
**Attention and Working Memory**					
Visual attention Test (/50)	45	9*	41	/	41
Trial Making Test					
- Test A sec.	38	n.a.	69	43	40
- Test B sec.	75	n.a.	167	150	129
- B-A sec.	37	n.a.	98	107	89
**Short-term memory**					
Digit Span Forward	6	/	6	/	6
Digit Span Backward	4	/	4	/	4
Corsi Block Span Forward	5	3*	4	/	5
Corsi Block Span Backward	6	2*	4	/	4
**Verbal long-term memory**					
Babcock (/28)	18,5	/	16	/	20,5
Associate learning (/22,5)	13,5	/	/	/	10
FCSRT-16-free					
- Tot. Immediate Free Recall (/48)	32	/	/	/	33
- Riev. Differed Free Recall (/16)	13	/	/	/	10
**Visuo-spatial long-term Memory**					
Rey – differed recall (/36)	19,5	9*	12	19	16,5
Visuo-spatial Supraspan					
- Immediate recall (/29,16)	11,18*	/	/	/	8,84*
- Differed recall (/1,62)	1,4	/	/	/	0*
**Visuo-spatial Functions**					
Rey Figure – Copy (/36)	34	12*	23,5*	33	24*
Judgment of Line Orientation Test (/30)	25	/	24	/	25
Clock Drawing Test (0/10)	/	4,5*	7	/	/
VOSP					

- Pre-test (/20)	18	/	/	/	20
- Incomplete Letters (20)	20	/	/	/	19
- Silhouettes (30)	23	/	/	/	22
- Object decision (/20)	16	/	/	/	16
- Progressive Silhouette (/20)	15	/	/	/	16
- Dot counting (/10)	5*	/	/	/	10
- Position Discrimination (/20)	20	/	/	/	18
- Number location (/10)	10	/	/	/	8
- Cube Analysis (/10)	10	/	/	/	8
**Language**					
Phonemic Fluency	55	/	49	/	39
Semantic Fluency	46	/	30	/	44
WAIS IV -Similarity (/36)	30	/	/	/	26
Visual Naming					
- Not Livings (/32)	32	/	32	/	32
- Livings (/32)	32	/	32	/	31
**Executive functions**					
Raven CPM 47 (/36)	31	n.a.	28	/	28
FAB (/18)	18	13*	16		15
Stroop					
- Time interference sec.	20	/	36,5	/	34
- Error interference (/30)	0	/	1	/	3
Time And Weight Estimation Task (STEP)					
- Time total (/30)	23	/	/	/	21
- Weight total (/30)	16*	/	/	/	23
- Time + Weight total (/60)	39*	/	/	/	44
- Time – Weight total (/60)		/	/	/	/
Modified Wisconsin Card Srting Test - MCST					
- Categories (/6)	4	/	/	/	6
- Errors (/47)	12	/	/	/	15
- Perseverative errors (/47)	4	/	/	/	4

Mental health screening					
Hospital Anxiety and Depression Scale - HADS					
- Anxiety (/21)	2	/	/	/	3
- Depression (/21)	3	/	/	/	2
Activities of Daily Living					
- ADL (/6)	6	/	/	/	6
- IADL (/5)	8	/	/	/	5

Abbreviations: Activities of daily living (ADL); Instrumental activities of daily living (IADL); frontal assessment battery (FAB)

Supplementary Table S4. Unilateral spatial hemineglect assessment in patient 2

**Test**	**post edema STN-DBS**	**6 weeks later**	**4 months later**
**Apples Cancellation Task**			
Left Omissions (/20)	20	1	0
Right Omissions (/20)	5	4	0
Accuracy (total omissions)	33*	8*	0
Egocentric Asymmetry (/20)	15*	3*	0
Total False positives	4	4	0
Allocentric Asymmetry (/20)	2*	4*	0
Total time (sec.)	300	146	130
**Line bisection test (/3)**	0*<br>Slight right shift	3<br>Normality	/
**Symbols cancellation test**			
Left Omissions (/15)	15	1	/
Rigth Omissions (/15)	7	0	/
Central Omissions (/5)	5	2	/
Total Omissions	27*	3	/
Omissions Difference (right-left)	8*	1	/
**Visual extinction**	Absent	/	/
**Letters cancellation test**			

Left Omissions (/53)	53	0	/
Rigth Omissions (/51)	32	0	/
Total Omissions	85*	0	/
Omissions Difference (right-left)	21*	0	/

Supplementary Table S5. Comparison between the different neuropsychological assessments (pre-surgical intervention, post-edema, follow-up) in patient 3

	**Pre-STN-DBS**	**Post-STN-DBS**	
**Test**	**16 months**	**1 week**	**6 months**
**Screening**			
Mini-Mental State Examination (/30)	28	21*	29
**Attention and Working Memory**			
Visual Attention Test (/50)	42	19*	42
Trial Making Test			
- Test A sec.	49	264*	44
**Short-term Memory**			
Digit Span Forward	5	5	5
Digit Span Backward	5	3	3
Corsi Block Span Forward	5	2*	4
Corsi Block Span Backward	5	2*	4
**Verbal Long-term Memory**			
Babcock (/28)	16	11,5	15,5
FCSRT (/16)	9	/	/
**Visuo-Spatial Long-term Memory**			
Rey Figure – differed recall (/36)	16	14	19
Visuo-spatial Supraspan			
- Immediate recall (/29,16)	21,07	/	/
- Differed recall (/1,62)	1,62	/	/
**Visuo-spatial Functions**			
Rey Figure - copy (/36)	33	24*	36
Clock DrawingTest (/10)		0*	7
VOSP			
- Pre-test (/20)	20	/	/
- Incomplete Letters(20)	18	/	/
- Silhouettes (30)	23	/	/
- Object Decision (/20)	14	/	/
- Progressive Silhouette (/20)	12*	/	/
- Dot counting (/10)	9	/	/
- Position Discrimination (/20)	20	/	/
- Number Location (/10)	10	/	/
- Cube Analysis (/10)	9	/	/
**Language**			

Phonemic Fluency	16	9*	19
Semantic Fluency	35	19*	35
Visual Naming			
- Not Livings (/32)	28	/	/
- Livings (/32)	31	/	/
**Executive functions**			
Raven CPM 47 (/36)	30	17*	27
FAB (/18)	16	9*	15
Stroop			
- Time interference sec.	22,5	49*	17
- Error interference (/30)	0	0	0
Modified Wisconsin Card Srting Test – MCST			
- Categories (/6)	6	6	/
- Errors (/47)	16	6	/
- Perseverative Errors (/47)	5	3	/
**Mental health screening**			
Hospital Anxiety and Depression Scale – HADS			
- Anxiety (/21)	14*	/	/
- Depression (/21)	10*	/	/

Abbreviations: Activities of daily living (ADL); Instrumental activities of daily living (IADL); frontal assessment battery (FAB).

Supplementary Table S6. Unilateral spatial hemineglect assessment in patient 3

Test	post edema STN-DBS	6 months later
**Apples Cancellation Task**		
Left Omissions (/20)	12	1
Right Omissions (/20)	9	3
Accuracy (total omissions)	28*	4
Egocentric Asymmetry (/20)	3*	2
Total False positives	0	0
Allocentric Asymmetry (/20)	0	0
Total time (sec.)	142	85
